# A new genus and species of native exotic millipede in Australia (Diplopoda, Polydesmida, Paradoxosomatidae)

**DOI:** 10.3897/zookeys.498.9716

**Published:** 2015-04-21

**Authors:** Robert Mesibov, Catherine A. Car

**Affiliations:** 1 Queen Victoria Museum and Art Gallery, 2 Invermay Road, Launceston, Tasmania 7248, Australia; 2 Department of Terrestrial Zoology, Western Australian Museum, Locked Bag 49, Welshpool DC, Western Australia 6986, Australia

**Keywords:** Diplopoda, Polydesmida, Paradoxosomatidae, New South Wales, Victoria, Tasmania, Australia

## Abstract

*Taxidiotisoma portabile* **gen. n.**, **sp. n.** is described from scattered populations in New South Wales, Victoria and Tasmania, Australia. Populations of *Taxidiotisoma portabile* in Victoria, Tasmania and parts of New South Wales occur in urban, suburban and agricultural areas, with no collections of the species in natural habitats in the same district. *Taxidiotisoma portabile* is likely to be a native exotic species whose home range is in eastern New South Wales.

## Introduction

We use the term “native exotic” for a species introduced and established well outside its native range, but still within its broader native region (Mesibov 2008, Car 2009). Three Australian paradoxosomatid millipedes clearly fit this description: *Akamptogonus novarae* (Humbert & de Saussure, 1869), *Heterocladosoma bifalcatum* (Silvestri, 1898) and *Solaenodolichopus pruvoti* (Brolemann, 1931).

Although its original range is still uncertain, *Akamptogonus novarae* is believed to be native to eastern Australia (Hoffman 1979). It occurs in urban and suburban areas in New South Wales, Tasmania, Victoria and Western Australia (locality records in Mesibov 2006–2015), and has been introduced to New Zealand (Rowe and Sierwald 2006), the Hawaiian Islands (Shelley and Lehtinen 1998) and San Francisco, California, in the United States (Hoffman 1979).

*Heterocladosoma bifalcatum* is likely to be native to the Brisbane area in southeast Queensland (Mesibov 2008), but it is now found in the Sydney metropolitan area (Rowe and Sierwald 2006) and elsewhere in New South Wales (Mesibov 2006–2015).

*Solaenodolichopus pruvoti* is also likely to be native to the Brisbane area (Mesibov 2014). It was first described from a town in New Caledonia and is now well established in the Perth metropolitan area in Western Australia, 3600 km from Brisbane (Mesibov 2014).

Here a new genus and species of Australian paradoxosomatid is described which we suspect is native to eastern New South Wales, but which has also been collected in urban, suburban and agricultural areas in New South Wales, Tasmania and Victoria.

## Materials and methods

“Male” and “female” in the text refer to adult individuals. In this paper, the labeling of the different structures on the gonopod mainly follows that of Car and Harvey (2013) for convenience, and is not intended to suggest homologies with podomeres nor, necessarily, with similarly labeled structures in other papers (Car and Harvey 2013, Car et al. 2013).

All specimens are stored in 75–80% ethanol in their respective repositories. Gonopod images were generated with a Leica MZ16A automontage imaging system using Leica Application Suite Version 3.7.0. Other photomicrographs are manually stacked composites, taken with a Canon EOS 1000D digital SLR camera mounted on a Nikon SMZ800 binocular dissecting microscope equipped with a beam splitter and processed with Zerene Stacker 1.04 software. Images were prepared for publication using GIMP 2.8. The locality map (Fig. 5) was prepared using ArcView 3.2 GIS.

Suppl. material 1 tabulates data for known specimen lots of the new species as of 30 March 2015 (data also available online in Mesibov 2006–2015). Locality details are given with latitude and longitude based on the WGS84 datum. Our estimate of the uncertainty for a locality is the radius of a circle around the given position, in metres or kilometres.

Abbreviations in text and Suppl. material 1 (all in Australia): AM = Australian Museum, Sydney; NMV = Museum Victoria, Melbourne; NSW = New South Wales; QVMAG = Queen Victoria Museum and Art Gallery, Launceston; Tas = Tasmania; Vic = Victoria.

## Results

### Order Polydesmida Pocock, 1887 Suborder Strongylosomatidea Brölemann, 1916 Family Paradoxosomatidae Daday, 1889 Subfamily Australiosomatinae Brölemann, 1916 Tribe Antichiropodini Brölemann, 1916

#### Genus Taxidiotisoma

Taxon classificationAnimaliaPolydesmidaParadoxosomatidae

Mesibov & Car gen. n.

http://zoobank.org/5730FE05-EB5B-4C0D-9A75-9E3984549968

##### Type species.

*Taxidiotisoma portabile* Mesibov & Car, sp. n., by present designation.

##### Other assigned species.

None.

##### Diagnosis.

In gonopod structure, *Taxidiotisoma* gen. n. is closest to *Antichiropus* Attems, 1911, *Australodesmus* Chamberlin, 1920, *Pogonosternum* Jeekel, 1965 and *Pseudostrongylosoma* Verhoeff, 1924 in the Australian paradoxosomatid fauna (see Remarks). Differs from *Antichiropus* in lacking a process on the lateral surface of the femorite, from *Pogonosternum* in having the distal portion of the acropodite divided into two rather than three branches, from *Pseudostrongylosoma* in having a divided solenomere, and from *Australodesmus* in having a Y-shaped solenomere rather than a flagellum-and-sheath solenomere.

##### Name.

Greek *taxidiotis*, “traveller” + *soma*, Greek “body’, often used as an ending for generic names in Paradoxosomatidae; neuter gender.

##### Remarks.

The gonopod of *Taxidiotisoma portabile* sp. n. appears most similar to that of species in *Antichiropus* Attems, 1911, *Australodesmus* Chamberlin, 1920, *Pogonosternum* Jeekel, 1965 and *Pseudostrongylosoma* Verhoeff, 1924, all four of which have been assigned to Antichiropodini by Jeekel (1968, 1979). In all five genera a long, well-demarcated femorite abruptly ends in several prominent processes, one of which is the solenomere. In *Antichiropus* there may be more than one non-solenomere process, but there is always one that arises on the lateral surface of the femorite; this lateral process is lacking in *Taxidiotisoma portabile* sp. n. In addition, *Antichiropus* species have a long, free solenomere that tends to spiral, whereas that of *Taxidiotisoma portabile* sp. n. is short and Y-shaped. In *Pogonosternum* species there are three acropodite branches, while in *Australodesmus*, *Pseudostrongylosoma* and *Taxidiotisoma* gen. n., there are only two, of more or less equal size. *Pseudostrongylosoma sjoestedti* Verhoeff, 1924 has an undivided solenomere. In *Taxidiotisoma portabile* sp. n. the solenomere is Y-shaped, i.e. divided into two sub-branches spaced well apart and not greatly different in size, while in *Australodesmus divergens* Chamberlin, 1920 the solenomere is divided into a thin, flagellum-like branch carrying the terminus of the prostatic groove and a much larger, flattened, cowl-like branch sheathing the thinner branch.

*Taxidiotisoma portabile* sp. n. is also characterized by a peculiar flattening of the head in lateral view, the result of depression of the clypeus.

#### Taxidiotisoma portabile

Taxon classificationAnimaliaPolydesmidaParadoxosomatidae

Mesibov & Car sp. n.

http://zoobank.org/E6EBEA22-31F1-4D27-BE78-B8DAD795FA52

Figs 1 , 2 , 3 , 4

##### Holotype.

Male, Munmorah State Reserve, NSW, 0.5 km along beach track opposite National Parks and Wildlife Service Station turnoff, site MUNI01/09, -33.2094 151.5894 ±25 m, pitfall 13–23 May 1998, L. Wilkie, AM KS.94041.

##### Paratypes.

2 males, 1 female, details as for holotype but 21 April - 1 May 1997, site MUNI01/10, AM KS.93366.

##### Other material.

100 males, 22 females and 5 juveniles (see Suppl. material 1 for details).

##### Description.

**Figure 1.**
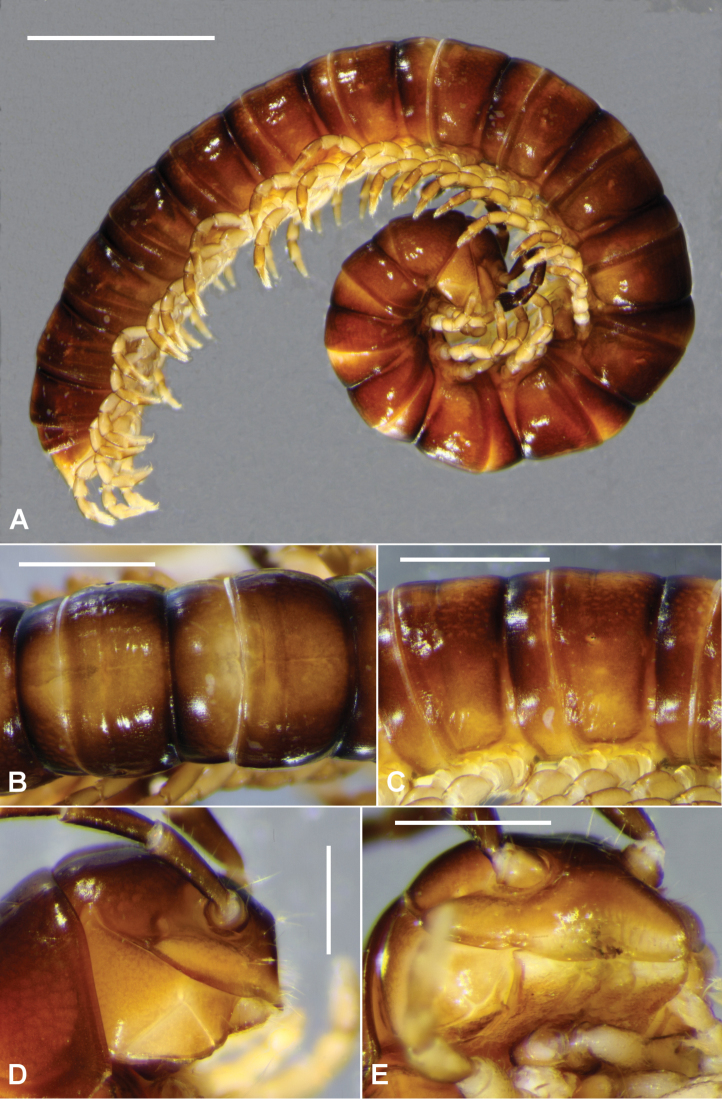
*Taxidiotisoma portabile* sp. n., male ex NMV K-12071. **A** Habitus **B** dorsal views of midbody rings **C** lateral views of midbody rings; anterior to right **D** Lateral views of head **E** oblique views of head. Scale bars: 2.5 mm (**A**); 1 mm (**B, C**); 0.5 mm (**D, E**).

Male/female approximate measurements: length ca 20/20 mm, maximum midbody width 1.5/1.8 mm. Body shiny (Fig. 1), colour in alcohol medium brown, dark brown either side of waist and dorsal portion of sides, dorsally with large, light brown patch spanning rear of metazonite and front of prozonite; antennae medium brown, darker distally; legs tan to pale brown, darker distally.

**Figure 2.**
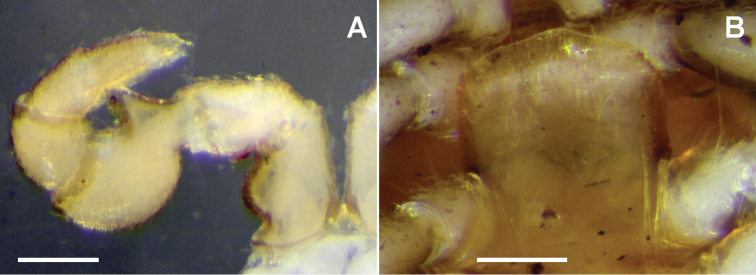
*Taxidiotisoma portabile* sp. n., male ex NMV K-12071. **A** Leg 1 **B** Sternal lamella on ring 5, posterior view. Scale bars: 0.2 mm.

Male with vertex and frons almost bare, clypeus sparsely setose; clypeus strongly depressed, head truncate in profile (Figs 1D, 1E); vertigial sulcus distinct, ending above level of antennal sockets; post-antennal groove moderately deep; antennal sockets separated by 1.3× socket diameter. Antenna slightly clavate, reaching dorsally only to rear of collum; antennomeres with relative lengths (2=3)>(4=5=6); 6 thickest but 5 and 6 subequal in apical width. Collum with subparallel anterior and posterior margins in dorsal view, strongly convex, lateral corner rounded. Head very slightly narrower than collum; collum to ring 18 nearly uniform in width, rings 2 and 3 slightly narrower. Ring 2 paranotum a thin, longitudinal ridge set low on ring, a little below collum corner; no paranota on other rings. No trace of pleural keels. Prozonites and metazonites (Fig. 1B, C) smooth, bare; transverse furrow at ca 2/3 metazonite length from waist, indistinct, not extending laterally as far as ozopores; waist very short, shallow, indistinctly sculptured with longitudinal ridges; limbus a narrow, thin, continuous sheet. Pore formula normal; ozopore very small, round, opening just above 1/2 ring height and just posterior to 1/2 metazonite length; slight bulging of ring around ozopore. Spiracles on diplosegments above and just anterior to leg bases; anterior spiracle ovoid, long axis subvertical, rim produced anterodorsally as rounded tab; posterior spiracle subtriangular, rim low; spiracular filters slightly emergent. Midbody sternites very sparsely setose, longer than wide, cross impressions subequal in width and depth; no cones or projections on any sternites. Midbody legs with relative podomere lengths (prefemur=femur)>tarsus>(postfemur= tibia); femur ca 1.2× as long as tarsus; anterior leg prefemora only very slightly swollen dorsally. Pre-anal ring sparsely setose; epiproct extending past anal valves, in dorsal view tapering and truncate, tip ca 1/4 width of pre-anal ring; hypoproct broadly paraboloid; spinnerets in rectangular array, wider than long. Leg 1 (Fig. 2A) with large, pointed process on medial femur surface, directed mediodistally and slightly anteriorly.

Gonopore small, round, opening on slight distomedial bulge of leg 2 coxa. Sternal lamella (Fig. 2B) wide, >90% of width between leg 4 bases on ring 5, strongly leaning anteriorly; lateral margins straight, vertical; corners rounded; ventral margin slightly raised medially. Dense brush setae on tibiae and tarsi of all legpairs except legpair 1 and last 2 legpairs; brush setae long, fine, curving distally.

**Figure 3.**
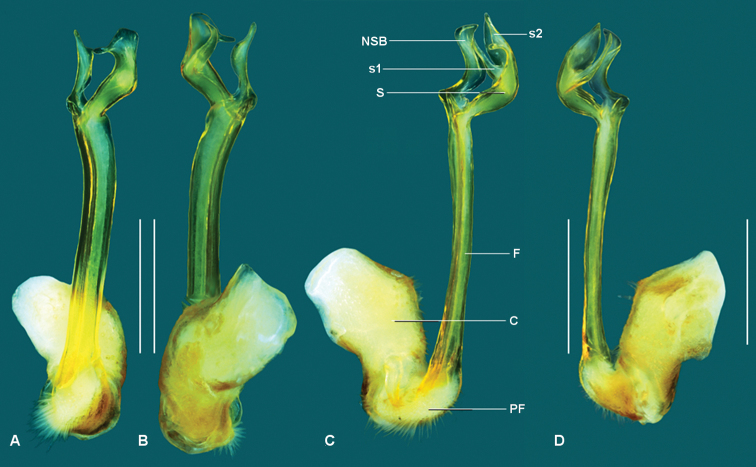
*Taxidiotisoma portabile* sp. n., holotype male (AM KS. 94041), left gonopod. **A** posterior **B** anterior **C** medial and **D** lateral views. Abbreviations: **C** coxa, **F** femorite, **NSB** non-seminiferous branch, **PF** prefemur, **S** solenomere, **s1** process with prostatic groove, **s2** cowl-shaped process. Scale bars: 0.5 mm.

**Figure 4.**
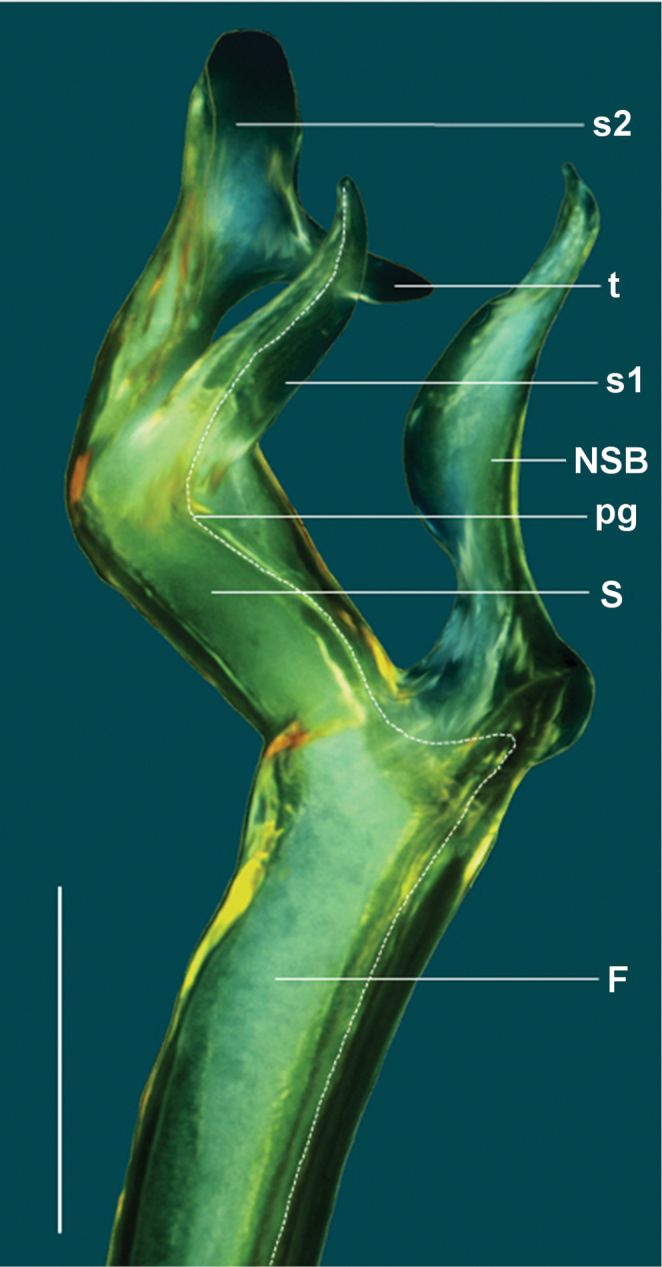
*Taxidiotisoma portabile* sp. n., holotype male (AM KS. 94041), detail of left gonopod tip, anterior view. Abbreviations: **F** femorite, **NSB** non-seminiferous branch, **S** solenomere, **s1** process with prostatic groove, **s2** cowl-shaped process, **pg** prostatic groove, **t** tooth. Dotted line denotes path of prostatic groove. Scale bar: 0.2 mm.

Gonopod aperture just wide enough to accommodate gonocoxae, ca 1/2 ring 7 prozonite width. Gonopod telopodites (Figs 3, 4) straight, parallel, reaching leg 6 bases when retracted; sternite between legpairs 6 and 7 excavate. Gonocoxa (**C**) robust, much thicker than femorite but shorter, ca 1/2 femorite length; prefemur (**PF**) ovoid, ca 1/3 femorite length, leading directly into femorite with no noticeable process at femorite base; femorite (**F**) ca 2/3 acropodite length, upright, cylindrical; non-seminiferous branch (**NSB**) slightly shorter than solenomere (**S**), curved, thickest mid-length with asymmetrical pointed tip and distinct “elbow” at base; **S** broad, proximal end as thick as femorite, curved, divided at mid-length into shorter, pointed process (**s1**) carrying prostatic groove, finger-like in anterior view, and longer, broader, cowl-shaped process (**s2**) carrying noticeable tooth (**t**) at about mid-length. Prostatic groove running straight along anteromedial surface of telopodite, looping slightly into base of **NSB** before touching base of **S**, then running on medial surface of **S** to tip of **s1** (Fig. 4).

Female with depressed clypeus, without leg modifications; epigynum not raised, nearly straight, ca 1/4 ring 2 width; cyphopods not examined.

##### Distribution.

**Figure 5.**
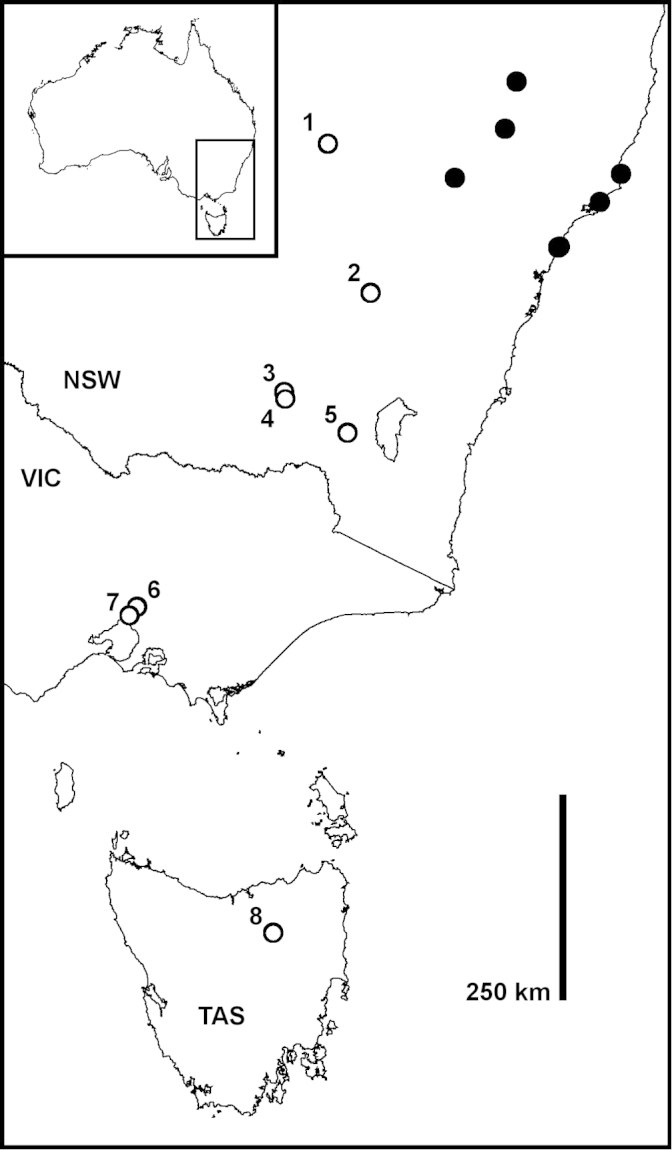
Known localities for *Taxidiotisoma portabile* sp. n. as of 30 March 2015 (filled and open circles). The eight numbered localities are discussed in the text. Geographic projection; inset shows location of main map.

*Taxidiotisoma portabile* sp. n. has been collected over a north-south range of ca 1000 km in eastern Australia (Fig. 5). A set of localities in eastern New South Wales (filled circles in Fig. 5) are in natural habitats in national parks or partly disturbed rangeland. Eight localities outside that set (unfilled circles in Fig. 5) are in cities, towns or long-cleared agricultural areas. These eight localities are numbered in Fig. 5 as follows:

1. A small area of riparian vegetation on the Macquarie River, surrounded by farmland;
2. The town of Cowra (collecting site not more exactly known), surrounded by farmland;
3. The campus of Charles Sturt University in the city of Wagga Wagga;
4. A small area of remnant native vegetation in the city of Wagga Wagga;
5. Disturbed native vegetation on a roadside adjoining a large artificial lake;
6. A recreation reserve in the suburbs of the city of Melbourne;
7. A park in the centre of the city of Melbourne;
8. Riparian parkland under a highway bridge in the town of Perth, surrounded by farmland.

Sampling in the areas surrounding and between these eight locations, both by the authors and by other collectors, has not yet yielded any specimens of *Taxidiotisoma portabile* sp. n. We therefore suspect that the species was introduced to these locations from its native range in eastern New South Wales.

##### Name.

Latin *portabilis*, “portable”, adjective. This species is almost certainly being transported to new areas in Australia by cars or trucks.

##### Remarks.

We do not know whether the eight “outlying” New South Wales, Tasmanian and Victorian samples (Fig. 5) represent long-established populations or ephemeral colonies, but the broad scattering of records suggests that this species will be found elsewhere in Australia in coming years.

## Supplementary Material

XML Treatment for Taxidiotisoma

XML Treatment for Taxidiotisoma portabile

## Supplementary materials

Supplementary material 1 Specimen records of Taxidiotisoma portabile Data type: Tab Separated Value File (tsv). Explanation note: Specimen records of Taxidiotisoma portabile as of 30 March 2015. This dataset is made available under the Open Database License (http://opendatacommons.org/licenses/odbl/1.0/). The Open Database License (ODbL) is a license agreement intended to allow users to freely share, modify, and use this Dataset while maintaining this same freedom for others, provided that the original source and author(s) are credited.Robert Mesibov, Catherine A. Car
